# A Machine Learning Approach to Predicting New-onset Depression in a Military Population

**DOI:** 10.1176/appi.prcp.20200031

**Published:** 2021-02-12

**Authors:** Laura Sampson, Tammy Jiang, Jaimie L. Gradus, Howard J. Cabral, Anthony J. Rosellini, Joseph R. Calabrese, Gregory H. Cohen, David S. Fink, Anthony P. King, Israel Liberzon, Sandro Galea

## Abstract

**Objective:**

Depression is one of the most common mental disorders in the United States in both civilian and military populations, but few prospective studies assess a wide range of predictors across multiple domains for new-onset (incident) depression in adulthood. Supervised machine learning methods can identify predictors of incident depression out of many different candidate variables, without some of the assumptions and constraints that underlie traditional regression analyses. The objectives of this study were to identify predictors of incident depression across 5 years of follow-up using machine learning, and to assess prediction accuracy of the algorithms.

**Methods:**

Data were from a cohort of Army National Guard members free of history of depression at baseline (*n* = 1951 men and 298 women), interviewed once per year for probable depression. Classification trees and random forests were constructed and cross-validated, using 84 candidate predictors from the baseline interviews.

**Results:**

Stressors and traumas such as emotional mistreatment and adverse childhood experiences, demographics such as being a parent or student, and military characteristics including paygrade and deployment location were predictive of probable depression. Cross-validated random forest algorithms were moderately accurate (68% for women and 73% for men).

**Conclusions:**

Events and characteristics throughout the life course, both in and outside of deployment, predict incident depression in adulthood among military personnel. Although replication studies are needed, these results may help inform potential intervention targets to reduce depression incidence among military personnel. Future research should further refine and explore interactions between identified variables.

Depressive disorders are among the most common mental disorders in both civilian and military populations in the United States (1,2). Major depressive disorder (MDD) was the second leading cause of disability in the United States in 2010 out of all medical conditions (3). Given this high burden, there is a need to identify characteristics of persons at high risk for developing depression, particularly in a military environment where soldiers may frequently deploy to high-stress situations andmay be more feasible to monitor and intervene on compared to most civilian populations.

The broad goal of supervised machine learning is optimized prediction, and it comprises algorithmic, data-driven approaches that can handle large numbers of predictor variables (4,5). In particular, classification tree and random forest classifiers construct nonparametric algorithms that promote visual inspection of the data and an understanding of complicated interactions and nonlinear associations that are more difficult to identify and interpret using other methods (4–7) or that would not otherwise be detected (8–10), allowing for identification of complex risk profiles without a priori hypotheses (7).

Among military populations, most studies investigating predictors of mental health problems have focused on military—and particularly deployment—experiences rather than a full range of characteristics and stressors occurring both in and outside of military service. A broader picture of risk is needed, particularly for part-time soldiers including the National Guard, who frequently transition between military and civilian life. Supervised machine learning methods can identify a wide array of factors associated with incident depression in this group.

Supervised learning has been used to predict psychiatric outcomes including suicide (11–13), posttraumatic stress disorder (PTSD) (14,15), depression in very specific groups (e.g., elderly populations; (16)), comorbid depression among patients with chronic physical conditions (17), and depression treatment response in clinical samples (18–20). To the best of our knowledge, classification trees and random forest have not yet been applied to predicting new-onset (incident) depression in a military population.

Our objectives for this study were to (a) use a range of potentially predictive characteristics and experiences from across the life course to discern which variables and their interactions predict incident depression, using classification tree and random forest algorithms, and (b) assess predictive accuracies of these algorithms using cross-validation, in a cohort of U.S. Army National Guard members.

## METHOD

### Data source

We used data from the Ohio Army National Guard Mental Health Initiative (OHARNG-MHI), an ongoing cohort study that began in 2008–2009. Details of recruitment are described elsewhere (21). This cohort—and the Ohio Army National Guard in general—is representative of the U.S. Army National Guard population as a whole in terms of many demographic and social factors such as military rank, gender, and age (21,22).

The first and primary cohort of the study (n = 2616 participants at baseline) completed telephone interviews approximately once per year for 6 years. The baseline interview assessed demographics, mental health disorders, military experiences, and potentially traumatic life events (“traumas,” e.g., major accidents, abuse) that occurred throughout the life course, whereas the follow-up interviews primarily assessed past-year events. In order to mitigate the loss of sample size over time and related changes in demographics due to attrition, smaller samples of newer recruits to the Guard replenished the original group of respondents each year, beginning in the third year of the study, creating a dynamic cohort study design (23). The analytic sample (1951 men and 298 women) included respondents from OHARNG-MHI who were present for at least one follow-up interview and had no history of depression at baseline (their first interview, regardless of the calendar year of entry into the study).

The Ohio National Guard and the institutional review boards of University Hospitals Case Medical Center, University of Toledo, University of Michigan, Ann Arbor Veterans Administration Medical Center, Columbia University, Boston University Medical Campus, and the Office of Human Research Protections of the U.S. Army Medical Research and Materiel Command approved this study protocol. Respondents provided verbal informed consent after receiving a complete description of the study.

### Outcome

Probable depression (henceforth referred to as “depression”) was measured with the Patient Health Questionnaire (PHQ-9) (24) and classified according to the Diagnostic and Statistical Manual for Mental Disorders version IV (DSM-IV) criteria. The construct was validated as part of the parent study, using a Structured Clinical Interview for DSM-IV-TR Axis I Disorders (non-patient version) in-person interview among a random subsample of 500 members of the original cohort (25). Any depressive disorder, which includes the DSM-IV categories of MDD and other depressive disorder, was used to define depression in this study due to higher sensitivity compared to MDD only (51% vs. 35%), without sacrificing specificity (83%), when validated against the in-person psychiatric interviews.

This definition corresponds to reporting a period of at least 2 weeks in the past year with two or more cooccurring symptoms, where one of the symptoms is depressed mood or anhedonia (inability to feel pleasure), with a frequency of “more than half the days” or “nearly every day.” Having thoughts of self-harm or suicide is an exception to the frequency criteria, counting as a symptom when reported at any frequency.

Incident depression was established by collapsing up to 5 years of follow-up data into one binary measure for each participant, to represent whether the individual had new-onset depression at any point during their follow-up.

### Predictors

In order to preserve temporality, all potential predictors were collected from the baseline interviews, with the exception of four adverse childhood experiences (ACEs) which were added to the study in the second year for the original cohort (but which were assessed at baseline for the following three cohorts).

Our set of a priori predictors included all questions or constructs (i.e., variables created from groups of questions or symptoms) from the baseline surveys, as long as the variables had at least five respondents per cell (category). There were 84 total potential predictors for men and 72 for women; women had fewer potential predictors due to their smaller sample (variables with less than five individuals per cell were either removed, or where possible, categories were combined).

These potential predictors included 12 demographic variables, seven military characteristic variables (e.g., rank), eight health-related variables (e.g., other mental health disorders, substance use), two social support variables, eight general life stressors (e.g., financial problems), and 47 traumas, including ACEs and also more recent traumas (e.g., witnessing death), both in and outside of deployment. All potential predictors and their prevalence in the sample are listed in Table SA1, and details of the predictors are provided in Appendix text.

### Statistical analysis

All analyses were gender stratified due to known differences in correlates of depression among men and women (26,27). We first ran single classification trees for each gender-specific sample using the *partykit* package in R, specifying no random variable selection at each node, minimums of 20 observations per split and 10 observations for the terminal nodes, and stop criterion based on univariate p values with a cutoff of p < 0.01. We plotted these trees in order to visually evaluate the data structure and identify key predictive variables and their interactions.

Next, we constructed 10-fold cross-validated random forests, which (a) consolidate across multiple classification trees to add random variation and avoid overfitting to any particular subsample, and (b) test and train the algorithms on different combinations of subsets of the sample. We used the *caret* and *RandomForest* packages with 1000 trees (28), five predictor variables randomly sampled at each node, and minimums of 20 observations per split and one observation for the terminal nodes. As described in Appendix, we tuned the algorithms to sample only from a subset of data for each tree (90% of true cases and an equal number of non-cases), in order to adjust for the class imbalance in our sample (29,30). Tuning this parameter provided better sensitivity compared to algorithms calculated from default methods.

Using the cross-validated predicted values, the average area under the receiver operating characteristic curve (AUC) was calculated for each gender-specific algorithm, in addition to the average sensitivity, specificity, and accuracy (the overall proportion of correctly classified individuals). We assessed and plotted variable importance using average decrease in accuracy for each variable, which represents the reduction in accuracy that would result if a variable were randomly permutated (29). All analyses are explained in detail in Appendix. For the tree classification, missing data were handled using surrogate splits (see Appendix). For the cross-validated random forests, a complete-case analysis was performed, as surrogate splits cannot be used across folds.

## RESULTS

### Men

**FIGURE 1.**
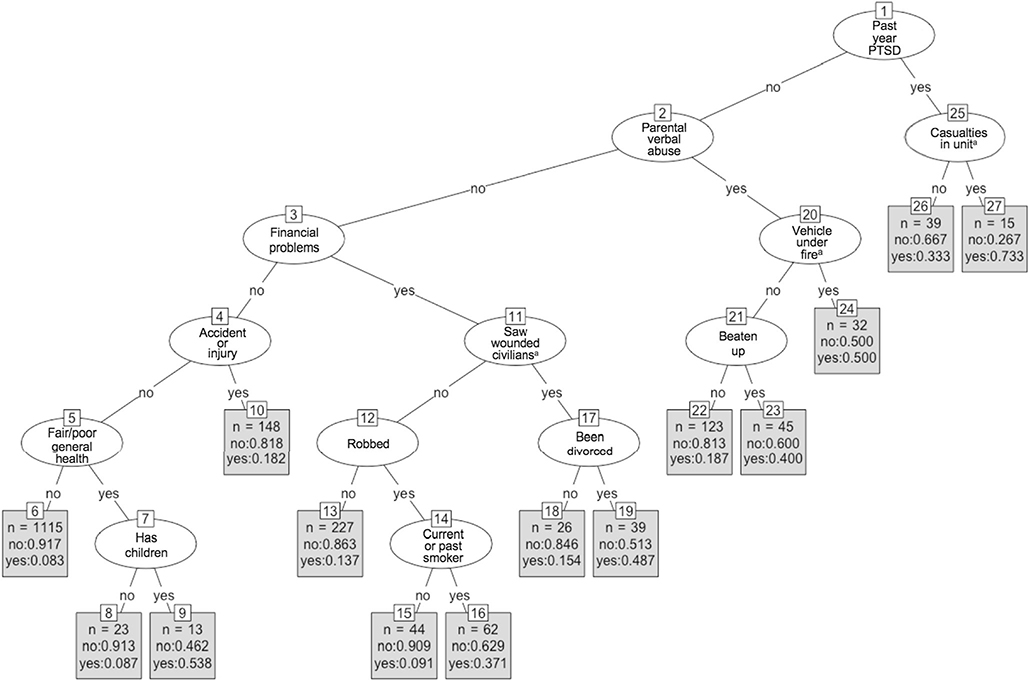
Classification tree for incident depression during follow-up among men (n = 1951)^a^ ^a^Predictors followed by the superscript “a” denote experiences that occured during the soldiers’ most recent deployment. PTSD, posttraumatic stress disorder. In gray boxes: n, number of individuals with selected combination of predictors; “no,” proportion without incident depression in this group; “yes,” proportion with incident depression in this group.

Incidence of depression over follow-up was 14.9% among men (other descriptive information is provided in the Appendix including Table SA1). Figure 1 shows the single classification tree among men. Past-year PTSD was the most predictive of depression overall. Among men with past-year PTSD, having had casualties in the unit with which they were most recently deployed was the next most predictive variable. Among those without past-year PTSD, parental verbal abuse in childhood (one of the ACEs) was next most important in predicting depression, and so on, down each branch. The combination of characteristics with the highest probability of incident depression was having both past-year PTSD and reporting a unit casualty during the most recent deployment (n=15, incidence=73.3%). The subgroup with the next highest incidence of depression (53.8%) included men who were parents or guardians of children under the age of 18 and who reported fair or poor general health compared to good or great health, but who reported no traumatic injuries/ accidents (other than transportation accidents), no financial problems, no childhood verbal abuse, and no past-year PTSD (n = 13).

**FIGURE 2.**
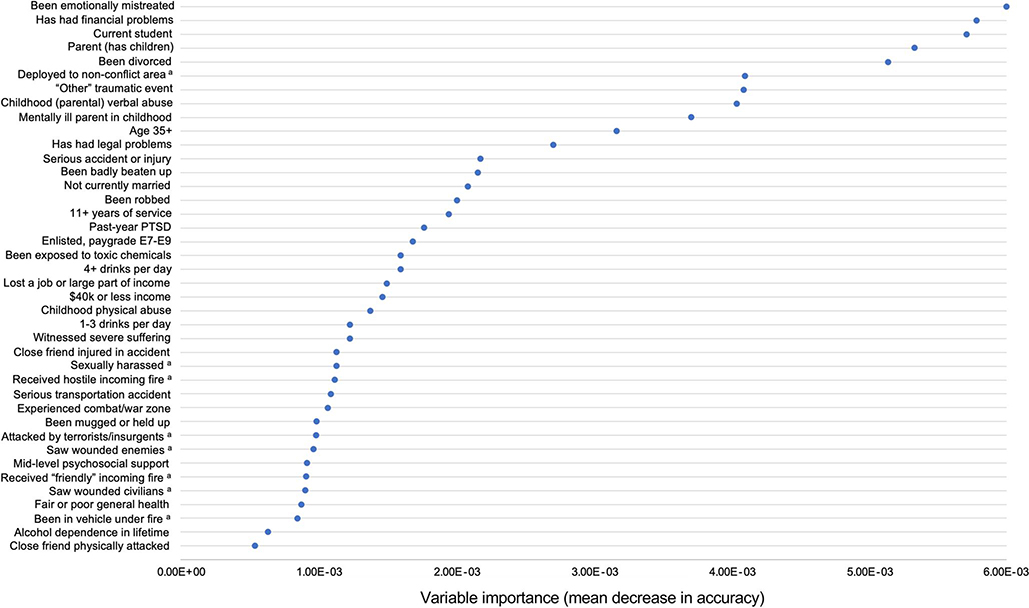
Variable importance plot from 10-fold cross-validated random forest for incident depression during follow-up, among men with no missing data (n = 1409)^a^ ^a^Predictors followed by the superscript “a” denote experiences that occured during the soldiers’ most recent deployment. PTSD, posttraumatic stress disorder.

Figure 2 shows a variable importance plot (of mean decreases in accuracy when each variable is removed) for the top 40 predictive variables among men from the cross-validated random forest (the values for all variables are listed in Table SA2). Reporting general life stressors (having been emotionally mistreated, financial problems, divorce); some demographic characteristics (being a current student, being a parent or guardian, being aged 35 or older); being deployed to a nonconflict area; and various traumas (including two ACEs) had the highest mean decreases in accuracy, meaning they were the top predictors. The cross-validated AUC and accuracy were 0.67% and 73.0%, respectively, with 46.8% sensitivity and 77.0% specificity when using the default threshold of predicted risk of 0.50.

### Women

**FIGURE 3.**
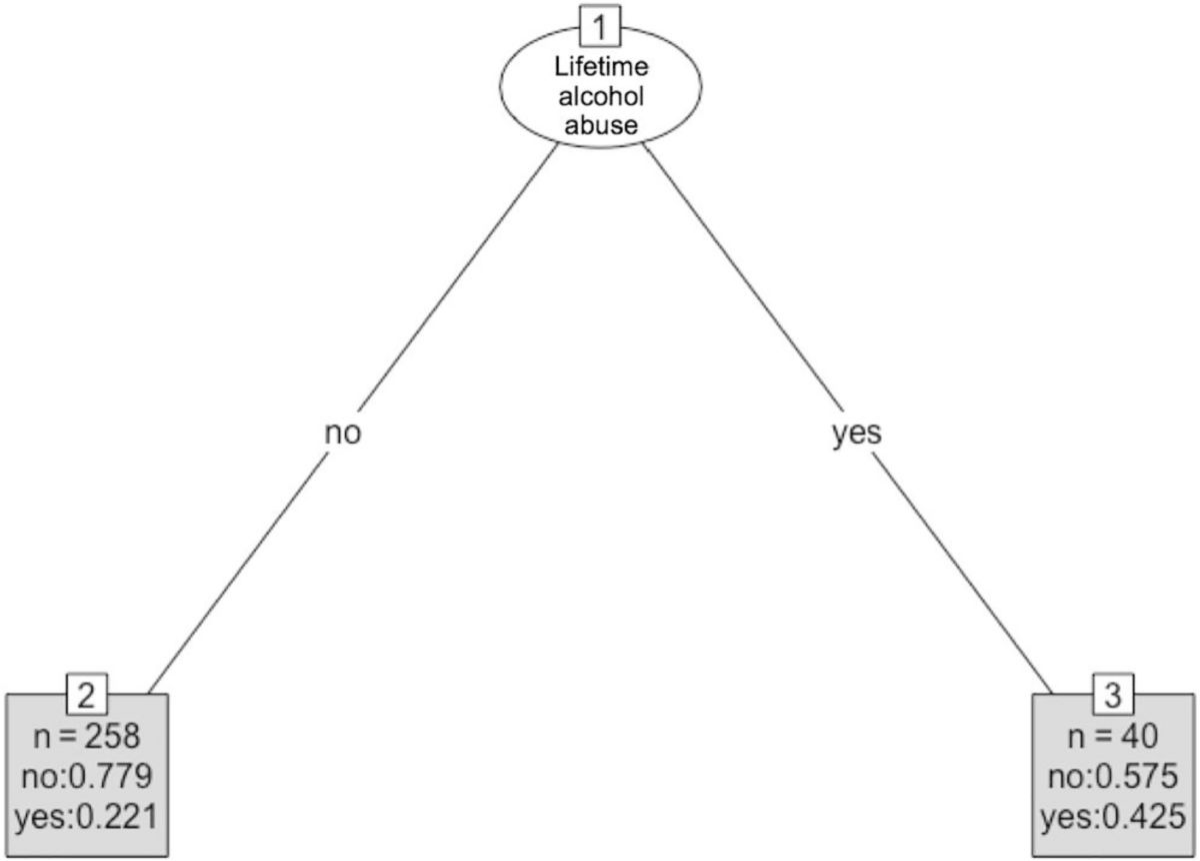
Classification tree for incident depression during follow-up among women (n = 298)^a^ ^a^In gray boxes: n, number of individuals with selected combination of predictors; “no,” proportion without incident depression in this group; “yes,” proportion with incident depression in this group.

Incidence of depression over follow-up was 24.8% among women. Figure 3 shows the single classification tree. Given the small sample size, only one split of the data was made, for alcohol abuse: women with a history of alcohol abuse at baseline had a 42.5% incidence of depression, whereas those who never had alcohol abuse had a 22.1% incidence of depression.

**FIGURE 4.**
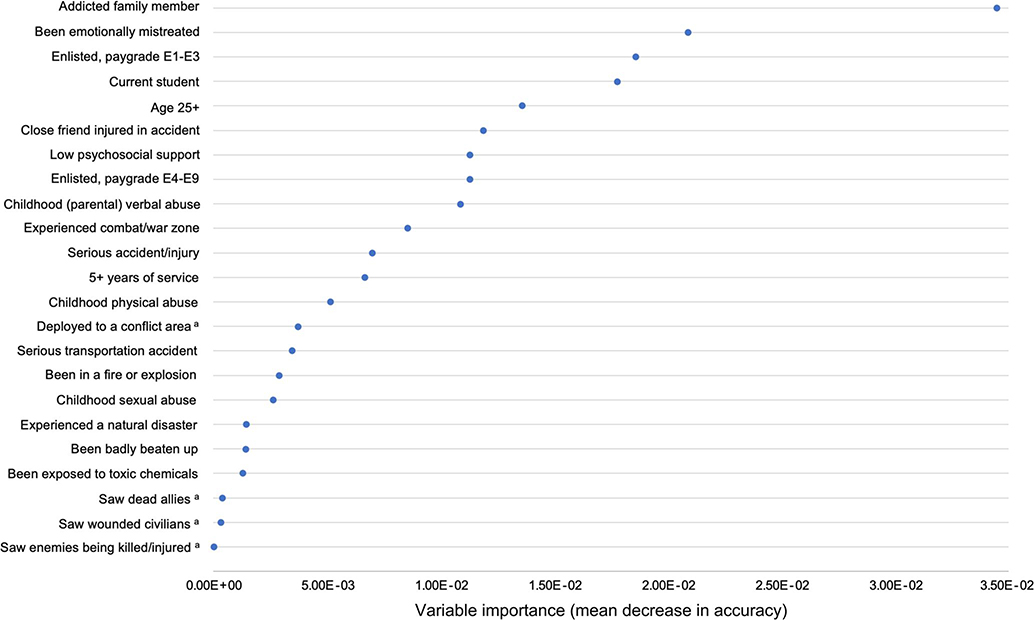
Variable importance plot from 10-fold cross-validated random forest for incident depression during follow-up, among women with no missing data (n = 251)^a^ ^a^Predictors followed by the superscript “a” denote experiences that occured during the soldiers’ most recent deployment.

Figure 4 depicts the variable importance plot for all predictive variables from the cross-validated random forest (these values are also listed in Table SA3). Life stressors (having a family member addicted to drugs or alcohol, having been mistreated); demographics (being enlisted with a relatively low paygrade, a student, and aged 25 or older); having a close friend or family member seriously injured in an accident other than a car accident; low psychosocial support; and childhood verbal abuse were among the top predictors. The cross-validated AUC was 0.67 and the average accuracy was 68.1%, with 75.3% specificity and 45.9% sensitivity when using the default threshold of predicted risk of 0.50.

## DISCUSSION

To the best of our knowledge, this study is the first to use classification trees and random forests to assess predictors of probable incident depression in a military sample. We found that, among both men and women, traumas and ACEs (particularly verbal abuse by a parent or guardian), stressors such as being emotionally mistreated, and demographics such as being a current student were predictive of incident depression during follow-up. Military characteristics (e.g., paygrade), low psychosocial support, and hearing about traumas happening to friends or family (e.g., a friend was in a serious accident) appeared more predictive of depression for women than for men, whereas PTSD, deployment location, personally experienced traumas (including combat-related experiences), and financial problems appeared more predictive among men compared to women.

Among men, recent deployment to a nonconflict area was predictive of depression, compared to being deployed to either a conflict area (Iraq or Afghanistan) or never having been deployed. This may be due to stressful and unexpected domestic deployments to areas affected by natural disasters—which have been increasing in recent years—or to areas of civil unrest after riots or massive protests, which can involve National Guard deployment. These types of domestic deployment may be more distressing for soldiers than combat deployments overseas, because they can involve confronting fellow citizens (e.g., at protests that become violent) or witnessing citizens suffer (in natural disaster contexts). This finding should be replicated, but it could indicate that additional resiliency training may be warranted for these unique deployment experiences. We were unable to compare incidence of depression by exact location or type of recent deployments, given small cells and lack of detailed questions on the surveys.

Past-year PTSD was the most predictive variable for incident depression among men in the single tree (and moderately predictive in the random forest, suggesting there may have been some overfitting in the single tree). This finding is broadly consistent with both with the only other study to use random forests to predict incident depression in a population-based sample (31) and with many non-machine learning studies that have consistently found comorbidity between PTSD and depression (32–35). The combination of having both past-year PTSD and reporting a unit casualty during the most recent deployment was particularly predictive of depression among men in the classification tree, for which the incidence of depression was 73.3%, or five times larger than the overall incidence of depression among men in this sample.

Among women in our study, PTSD was not predictive of incident depression, but lifetime PTSD status was included in the algorithm instead of past-year status, given the small number of women with PTSD in the past year in an already-small sample of women. This may be the reason why PTSD was not selected among women as being highly predictive, since history of PTSD may have occurred many years before onset of depression, and thus not as clinically or statistically relevant.

Our findings that ACEs and more recent traumas and stressors were predictive of incident depression (for both men and women) is also consistent with a prior machine learning study (31) as well as many non-machine learning studies that have modeled incident or prevalent depression with similar types of events as exposures or predictors (36–38). Traumas and stressors such as being mistreated have long been known to associate with depression outcomes (39–42), particularly when they occur during childhood, while brain is still developing (42,43).

Finally, our findings on financial problems, being a student, being of lower paygrade, and having children may all be related to financial stress, debt, and concern about being able to provide for one’s family, which have been found in non-machine learning contexts to be associated with depression (44–47).

Based on 10-fold cross-validation, our random forest algorithms were moderately accurate overall (73% accuracy for men and 68% for women). These values are in line with other studies predicting depression outcomes; Kautzky and colleagues (18), who used random forests to predict treatment-resistant depression, found accuracies of 68%–75%. Similarly, Jin and colleagues (17), who used four different prediction methods including random forests to model depression (also measured using the PHQ-9) among patients with diabetes, found comparable levels of accuracy (approximately 73%).

Limitations of our study include the use of baseline information alone to predict incident depression over follow-up. It follows that we lack (a) time-varying information assessed on the follow-up surveys that could be temporally closer to onset of depression compared to baseline variables, and (b) information on exact timing of prior events and experiences, as the baseline surveys primarily assessed events that occurred at some point in the past, without asking detailed information on timing (with the exception of other mental disorders). However, using only baseline predictors in this study established temporality between our predictors and outcome—a crucial aspect of valid prediction.

Another limitation is our use of the PHQ-9 for measuring depression. Although the PHQ-9 has been validated against a gold standard depression measure within this cohort as well as in many other populations (24,25), it is primarily a screening tool and was not designed as a diagnostic test. Thus, it is possible that there are individuals in this study with incorrectly classified depression status, which could have affected which variables were chosen as being predictive. Future studies should aim to replicate these results using diagnostic measures of depression.

Finally, we used a complete case analysis for the cross-validated random forests. Missing data in this study stem primarily from the fact that ACEs were not asked on the baseline survey for the first (and largest) cohort of participants. For those individuals, the ACEs were assessed in the second wave of the study, at which not all respondents were present. A smaller portion of missing data came from responses of “don’t know” or declining to answer questions such as income. As this is a prediction study and thus we are not aiming to isolate and measure the effect of any particular variable on depression, missing data are not as problematic of an issue as in an explanatory study. Generally, missing data among predictors in prediction modeling are thought to only create bias if missingness is related to the outcome variable (9,48). We have no reason to believe that this is the case in our study, as all predictors are from the baseline interview, at which time the outcome had not yet occurred (with the exception of the four ACEs assessed at Wave 2 for the primary cohort, which were missing by design, not by refusal to answer).

Despite these limitations, these results may help inform potential screening interventions for depression in this population. Algorithms represent concrete ways officials might identify characteristics associated with high risk of developing outcomes, regardless of underlying causal relationships; this might be especially useful in a military setting given that military personnel are feasible to monitor. For example, the REACH Vet algorithm, built by researchers using machine learning, helped the U.S. Department of Veterans’ Affairs to identify veterans at high risk for suicide (49,50), as part of a crucial undertaking at a time when suicides among military personnel have been increasing.

Future analytic work that aims to predict depression—preferably using larger samples and more specifically timed predictors than we were able to utilize in this study—should aim to replicate our findings and further refine interactions between variables identified here. Machine learning might also be used to predict particular subtypes of depression, given that the overall disorder is heterogeneous and takes on different forms in different individuals; this may improve prediction accuracy. Predictive accuracies of the algorithms could also be compared with individual-level prediction using more traditional types of regressions, or using other types of machine learning algorithms, including ensemble methods such as Super Learner which average across different types of algorithms. Finally, broader environmental and context-level variables—like unit-level characteristics in a military study or residential neighborhood-level characteristics in a general population survey—may be important for prediction of individual incident depression (51,52), and should be included as predictors in future studies, where sampling designs allow.

## Supplementary Material

Appendix

## HIGHLIGHTS

- In a cohort study of U.S. Army National Guard personnel, we found that stressors and traumas such as emotional mistreatment and adverse childhood experiences, demographics such as being a parent or student, and military characteristics including paygrade and deployment location were predictive of new-onset depression.
- Cross-validated random forest algorithms were moderately accurate for predicting depression.
- Among military personnel, events and characteristics throughout the life course—in and outside of deploy-ment—predict new-onset depression in adulthood.
