# Supplementary material for: A Machine Learning Approach to Predicting New-onset Depression in a Military Population
Source: Psychiatr Res Clin Pract. Author manuscript; Available in PMC 2021 Nov 2. (PMC8562467; doi:10.1176/appi.prcp.20200031)
Supplement: Appendix [file NIHMS1708818-supplement-Appendix.docx]

**Appendices:**

**Description of potential predictors in algorithms:**

The potential predictors included the following general categories (detailed in Table SA1): demographics, military characteristics, health-related variables, potentially traumatic events from the respondent’s most recent deployment as assessed by the Deployment Risk and Resilience Inventory (DRRI) (1), additional potentially traumatic events from the Life Events Checklist-Civilian Version (2) and the Detroit Area Survey of Trauma (3) (regardless of whether or not they occurred during deployment), individual traumatic events specific to childhood (four out of seven questions from the Adverse Childhood Experiences (ACEs) study (4)), individual stressful experiences that could occur at any point during the lifecourse (e.g., divorce, having serious financial trouble), responses to a psychosocial support and resources scale (six items adapted from the DRRI (1) which were summed and tertiled given the importance of relative differences in perceived social support among individuals in relation to mental health outcomes found in prior work using this cohort) (5), and the following Diagnostic and Statistical Manual for Mental Disorders, version IV (DSM-IV) mental disorders (DSM-5 criteria was not yet available at the time of the baseline interviews for the majority of cohorts): Generalized Anxiety Disorder (GAD, measured using the GAD-7 (6) with a cutoff score of 10 or a reported doctor’s diagnosis, and with a timescale of any point in lifetime for women and any point in the past year for men given their larger sample), posttraumatic stress disorder (PTSD; measured using the PTSD Check List-Civilian version (7) or a reported doctor’s diagnosis; lifetime for women and past-year for men), and both lifetime and past-year (tested separately, to avoid multicollinearity) alcohol abuse and alcohol dependence, included as separate alcohol use disorders according to the DSM-IV, assessed using the Mini‐International Neuropsychiatric Interview (8).

All variables were operationalized as categorical variables. Most predictors were inherently categorical, (e.g., trauma experience, location of most recent deployment), but variables that were originally continuous, such as age or years of military service, were coded into categories based on their distribution (e.g., split into tertiles or quartiles), as listed in Table SA1. All variables were entered into the algorithms as binary dummy variables. (For a variable with n different categories, n-1 dummy variables were entered, with one category left out as a reference group.) The reason for this categorization and use of dummy variables is that if some predictor variables have more categories than others (including continuous variables which would be treated by classification trees and random forests as having as many categories as they do values), they would automatically be retained by the algorithms more often than those with fewer categories, due to greater variation in the outcome variable by category of the predictor, which obscures the true predictive ability of each variable (9).

Since these variables were from the baseline interviews, they primarily describe events throughout the lifecourse without reference to specific timing (designed in order to keep the baseline interview relatively short), but some critical constructs were asked with reference to specific timing, such as mental disorder symptoms. As recency is likely relevant for prediction, we included past-year baseline constructs where available and where sample size allowed (e.g., we included past-year PTSD for men, but only included more general lifetime PTSD for women (still measured at baseline), due to the small number of women with past-year disorder).

**Description of tree classification, random forests, and cross-validation**:

*Classification trees*

Classification trees (also known as “decision trees”) choose key predictive variables from the larger group of potential predictors using the probabilities of the known outcome, in order of magnitude of bivariate associations (10). Trees are constructed by making repetitive partitions of the data—called splits—by such predictive variables, in order to form a hierarchical structure of nodes (variables or combinations of variables), which also serve as decision rules for the algorithm to predict the outcome (10–12). When creating these splits, the tree classification method uses “surrogate” splits for respondents who are missing data on predictors, based on variables other than the one used for the primary split (11). The ultimate goal of the classification tree is to end up with groups of observations that are relatively homogenous with respect to the outcome, at the terminal nodes (the end of the branches) (13).

*Random forests and cross-validation*

A Random forest can be conceptualized as an ensemble prediction method, consolidating across multiple decision trees, to avoid overfitting to any particular subsample. By combining different trees, the random forest capitalizes on the fact that individual trees can be unstable by themselves, but tend to produce the right prediction, on average (14). In random forests, the classification tree analysis (as described above) is generated on many different “training sets” or bootstrapped samples of the data (drawn with replacement), and tested on the observations not included in the training sample (also referred to as the “out of bag” sample) (13). Each tree within the random forest uses a different bootstrap sample and testing sample, and then the results are combined in order to produce an aggregated final result with less overall variance and better prediction than a single classification tree (9,15,16). As Bi and colleagues explain, when the trees are combined, they can “borrow strength” from one another, in order to achieve higher overall average predictive accuracy (12).

Cross-validation is performed to evaluate whether the random forest may be generalizable to an independent dataset (17). This is particularly important given that in general, a model trained and tested on the same sample tends to be overoptimistic (17,18). In cross-validation, the data are randomly split into ten subsets, or folds. A random forest is then generated—as described above—using nine of the folds and tested on the one remaining fold (which is considered an independent sample). This process is repeated over all combinations of folds, while computing the prediction accuracy each time. Finally, these estimates are averaged together across all iterations (13).

We ran the 10-fold cross-validations on non-missing data only, given that the surrogate split method for missing data cannot be applied to cross-validation as it can in single classification trees. We first ran the cross-validation with all possible predictor variables included, and then removed a few variables that contributed negatively to prediction (i.e., had a negative variable importance, meaning the model was more accurate without them) one at a time, in a stepwise fashion, until there were either no remaining negative values or the performance metrics were maximized.

To address the class imbalance in our sample (more non-cases than cases), we instructed the algorithm to sample from 90% of true cases (the maximum number of cases that could be chosen for the training phase, given that 10% of the sample is always saved for the testing phase), and an equal number of non-cases, in order to have the same number of true cases and true non-cases when building each tree. If this parameter is not tuned, the algorithm tends to do a better job of predicting the dominant class (14,19), which in this sample is being a non-case. For the sample of men (n = 1,409 total non-missing observations), 171 cases and 171 non-cases were sampled at each node. For the women (n = 251 total non-missing observations), 54 cases and 54 non-cases were sampled at each node.

Important predictors from random forests are compared using the average improvement in classification accuracy that each variable achieved across all different trees in the forest (13). In order to determine importance, the prediction accuracy of each tree is computed with each variable of interest and then again with a version of that variable that is randomly permutated (i.e., removing any true predictive power of that variable), and the difference in accuracy between these two versions is averaged across every tree (15,20). This is repeated for each variable, and the average differences in accuracy for all variables across all trees and folds are then plotted in order of magnitude, or variable “importance” (see Figures 2 and 4). The values of variable importance are relative to each other; large breaks between variables in the plot represent large relative differences in prediction; the numbers are not meaningful in isolation or when comparing across studies. In the variable importance plots in Figures 2 and 4, the predictors shown had a positive average decrease in classification accuracy (when removed from the trees) after the cross-validation.

*Descriptive information about sample*

As can be seen in Table SA1, about 41% of men were between the ages of 18 and 24; 29% were between the ages of 25 and 34; and 30% were older than 35. The women were younger overall, with 57.4% between the ages of 18 and 24. There was a very small proportion of individuals who identified as Hispanic overall, and only 6.8% of men were black while 4.4% identified as being of a race other than white or black. Slightly more than 37% of men had an annual income of $40,000 or less, while almost half of women reported that same income category. Table SA1 lists the prevalence for all demographics and other characteristics of the sample by gender.

**Table SA1. Prevalence of all predictors entered into algorithms, by category and stratified by gender.**

|  | **Men (n = 1,951)** | | **Women (n = 298)** | |
| --- | --- | --- | --- | --- |
|  | **n** | **%** | **n** | **%** |
| **Demographics** |  |  |  |  |
| Age 25-34 | 566 | 29.01 | 127 | 42.62 |
| Age 35+ (ref = age 18-25) | 587 | 30.09 |  |  |
| Hispanic ethnicity | 53 | 2.72 | N <5 | - |
| Black race | 133 | 6.83 | 61 | 20.47 |
| “Other” race (ref = white) | 85 | 4.37 |  |  |
| $40,000 or less annual income | 711 | 37.32 | 138 | 48.76 |
| $41,000-$80,000 annual income (ref = more than $80,000) | 742 | 38.95 | 90 | 31.80 |
| Not currently married | 1068 | 54.77 | 243 | 81.54 |
| High school or less education | 546 | 27.99 | 80 | 26.85 |
| Some college education (ref = more than college education) | 924 | 47.36 | 147 | 49.33 |
| Parent or dependent of someone younger than 18 | 769 | 39.42 | 64 | 21.48 |
| Current student | 561 | 28.83 | 135 | 45.30 |
| **Military characteristics** |  |  |  |  |
| 2-4 total years of service in U.S. military (for women) | - | - | 107 | 35.91 |
| 5+ total years of service in U.S. military (for women; ref = 0-1 years) | - | - | 105 | 35.23 |
| 3-10 total years of service in U.S. military (for men) | 659 | 33.81 | - | - |
| 11+ total years of service in U.S. military (for men; ref = 2 or fewer years) | 648 | 33.25 | - | - |
| Enlisted, paygrade E1-E3 | 508 | 26.12 | 109 | 36.70 |
| Enlisted, paygrade E4-E6 | 924 | 47.51 | 150 | 50.51 |
| Enlisted, paygrade E7-E9 (ref = Officer or Warrant Officer) | 204 | 10.49 |  |  |
| Deployed to an area of conflict during most recent deployment | 515 | 26.51 | 36 | 12.08 |
| Deployed to an area of NON-conflict during most recent deployment (reference = never deployed) | 556 | 28.62 | 64 | 21.48 |
| **Health-related variables** |  |  |  |  |
| Fair or poor self-rated general health (ref = good or great) ^a^ | 97 | 4.98 | 70 | 23.57 |
| Current or former smoker (ref = never smoked) | 1065 | 54.64 | 154 | 51.68 |
| 1-2 drinks per day on average days of drinking in past month (for women) | - | - | 98 | 32.89 |
| 3+ drinks per day on average days of drinking in past month (for women; ref = no drinking in past month) | - | - | 82 | 27.52 |
| 1-3 drinks per day on average days of drinking in past month (for men) | 811 | 41.98 | - | - |
| 4+ drinks per day on average days of drinking in past month (for men; ref = no drinking in past month) | 593 | 30.69 | - | - |
| DSM-IV alcohol dependence in lifetime | 372 | 19.07 | 24 | 8.05 |
| DSM-IV alcohol abuse in lifetime | 479 | 24.55 | 40 | 13.42 |
| DSM-IV PTSD (in past-year for men; in lifetime for women) | 54 | 2.77 | 23 | 7.72 |
| DSM-IV GAD (in past-year for men; in lifetime for women) | 24 | 1.27 | 25 | 8.71 |
| Low tertile of psychosocial support score | 655 | 33.83 | 103 | 34.68 |
| Middle tertile of psychosocial support score (ref= top tertile) | 736 | 38.02 | 81 | 27.27 |
| **Adverse childhood experiences (ACEs)** |  |  |  |  |
| Verbal abuse by a parent or other adult in home | 158 | 9.34 | 30 | 11.81 |
| Physical abuse by a parent or other adult in home | 147 | 8.69 | 27 | 10.63 |
| Sexual abuse by a parent or other adult in home | 10 | 0.59 | 12 | 4.72 |
| Parent or other adult in home was mentally ill | 166 | 9.81 | 38 | 15.02 |
| **Stressful events in lifetime** |  |  |  |  |
| Lost a job or a large part of income | 613 | 31.42 | 69 | 23.15 |
| Been divorced | 779 | 39.93 | 149 | 50.00 |
| Been emotionally mistreated | 404 | 20.73 | 94 | 31.54 |
| Had legal problems (e.g., have been sued) | 329 | 16.88 | 48 | 16.11 |
| Been unemployed for 3 months or more | 670 | 34.38 | 92 | 30.87 |
| Had financial problems | 519 | 26.62 | 64 | 21.55 |
| Been robbed or had your house broken into | 495 | 25.37 | 60 | 20.13 |
| Had a family member with a serious drug or alcohol problem | 166 | 9.81 | 165 | 55.37 |
| **Traumatic events in lifetime (in or outside of deployment)** |  |  |  |  |
| Experienced combat or a war zone | 740 | 37.99 | 48 | 16.11 |
| Sexually assaulted or raped | 76 | 3.90 | 85 | 28.52 |
| Been in a fire or explosion | 536 | 27.52 | 27 | 9.06 |
| Been shot of stabbed | 138 | 7.08 | N <5 | - |
| Been kidnapped or tortured | 5 | 0.26 | N <5 | - |
| Been mugged or held up | 556 | 28.53 | 29 | 9.73 |
| Been badly beaten up | 272 | 13.95 | 15 | 5.03 |
| Been in a serious transportation accident | 486 | 24.91 | 67 | 22.48 |
| Been in another type of serious accident or injury | 305 | 15.67 | 29 | 9.73 |
| Experienced a natural disaster in which you were hurt or property was damaged | 284 | 14.56 | 37 | 12.42 |
| Been diagnosed with a serious illness | 123 | 6.31 | 19 | 6.40 |
| Your child was diagnosed with a serious illness | 43 | 2.21 | N <5 | - |
| Witnessed serious injury or death | 769 | 39.52 | 39 | 13.09 |
| Unexpectedly discovered a dead body | 344 | 17.65 | 21 | 7.05 |
| A close friend or family member was sexually assaulted | 615 | 31.55 | 134 | 45.12 |
| A close friend or family member was physically attacked | 535 | 27.42 | 80 | 26.85 |
| A close friend or family member was hurt in a serious transportation accident | 996 | 51.10 | 143 | 47.99 |
| A close friend or family member was hurt in another kind of accident | 571 | 29.30 | 85 | 28.62 |
| Experienced the sudden, unexpected death of a loved one | 1228 | 63.04 | 191 | 64.09 |
| Injured or killed someone else | 180 | 9.34 | N <5 | - |
| Witnessed severe human suffering | 566 | 29.13 | 44 | 14.77 |
| Had a serious operation | 245 | 12.57 | 34 | 11.45 |
| Been exposed to toxic substances or chemicals | 407 | 21.41 | 29 | 9.80 |
| Had another type of traumatic event | 372 | 19.09 | 56 | 18.79 |
| **Traumatic events that happened specifically during most recent deployment ^b^** |  |  |  |  |
| Encountered land/water mines or booby traps | 362 | 18.69 | 17 | 5.70 |
| Received hostile incoming fire from small arms, artillery, rockets, mortars, bombs, or IEDs | 570 | 29.37 | 39 | 13.09 |
| Received “friendly” incoming fire from small arms, artillery, rockets, mortars, bombs, or IEDs | 123 | 6.38 | 10 | 3.39 |
| Been in a vehicle under fire | 331 | 17.05 | 13 | 4.36 |
| Attacked by terrorists, insurgents, or civilians | 465 | 24.16 | 27 | 9.12 |
| Engaged in a battle with casualties in unit | 195 | 10.05 | 19 | 6.38 |
| Witnessed allies being seriously wounded or killed | 235 | 12.09 | 8 | 2.68 |
| Witnessed enemies being seriously wounded or killed | 222 | 11.42 | 10 | 3.36 |
| Fired weapon at enemy | 231 | 11.89 | N <5 | - |
| Killed or think you killed someone in combat | 129 | 6.83 | N <5 | - |
| Saw enemy soldiers after they had been severely wounded or disfigured | 243 | 12.51 | 11 | 3.69 |
| Saw bodies of dead enemies | 230 | 11.86 | 7 | 2.36 |
| Saw civilians after they had been severely wounded or disfigured | 262 | 13.51 | 18 | 6.04 |
| Saw bodies of dead civilians | 200 | 10.31 | 16 | 5.37 |
| Saw allies after they had been severely wounded or disfigured | 294 | 15.13 | 13 | 4.36 |
| Saw bodies of dead allies | 189 | 9.72 | 7 | 2.35 |
| Injured or wounded in combat | 38 | 1.95 | N <5 | - |
| Sexually harassed (during most recent deployment) | 173 | 8.88 | 50 | 16.84 |
| Head injury (during most recent deployment) | 43 | 2.22 | N <5 | - |

^a^ Good, fair or poor health for women (vs. very good or excellent).

^b^ Respondents who had never been deployed at baseline have a 0 for these variables.

Ref = reference group when entered into algorithms.

PTSD = posttraumatic stress disorder.

GAD = generalized anxiety disorder.

IED = improvised explosive device.

Percentages calculated among non-missing values.

**Table SA2. Mean decrease in accuracy of predictors from 10-fold cross-validated random forest among men with no missing data (n = 1,409).**

| **Predictor** | **Mean decrease in accuracy** |
| --- | --- |
| Been emotionally mistreated | 6.00E-03 |
| Has had financial problems | 5.78E-03 |
| Current student | 5.71E-03 |
| Parent (has children) | 5.33E-03 |
| Been divorced | 5.14E-03 |
| Deployed to an area of non-conflict ^a^ | 4.10E-03 |
| "Other" traumatic event | 4.09E-03 |
| Childhood (parental) verbal abuse | 4.04E-03 |
| Parent mentally ill in childhood | 3.71E-03 |
| Age 35+ | 3.17E-03 |
| Has had legal problems | 2.71E-03 |
| Serious accident/injury (other) | 2.18E-03 |
| Been badly beaten up | 2.16E-03 |
| Not currently married | 2.09E-03 |
| Been robbed | 2.01E-03 |
| 11+ years of service | 1.95E-03 |
| Past-year PTSD | 1.77E-03 |
| Enlisted, paygrade E7-E9 | 1.69E-03 |
| Been exposed to toxic substances | 1.60E-03 |
| 4+ drinks per day | 1.60E-03 |
| Lost a job or a large part of income | 1.50E-03 |
| $40k or less income per year | 1.47E-03 |
| Childhood physical abuse | 1.38E-03 |
| 1-3 drinks per day | 1.23E-03 |
| Witnessed severe human suffering (ever) | 1.23E-03 |
| Close friend injured in [other] accident | 1.13E-03 |
| Sexually harassed ^a^ | 1.13E-03 |
| Received hostile incoming fire ^a^ | 1.12E-03 |
| Serious transportation accident | 1.09E-03 |
| Experienced combat/war zone | 1.07E-03 |
| Been mugged or held up | 9.89E-04 |
| Attacked by terrorists, insurgents, or civilians ^a^ | 9.84E-04 |
| Saw wounded enemies ^a^ | 9.65E-04 |
| Mid-level psychosocial support | 9.17E-04 |
| Received "friendly" incoming fire ^a^ | 9.11E-04 |
| Saw wounded civilians ^a^ | 9.04E-04 |
| Fair or poor self-rated general health | 8.78E-04 |
| Been in a vehicle under fire ^a^ | 8.48E-04 |
| Alcohol dependence in lifetime | 6.34E-04 |
| Close friend physically attacked | 5.40E-04 |
| Experienced natural disaster | 5.27E-04 |
| Injured or killed someone else | 5.14E-04 |
| Saw dead civilians ^a^ | 5.13E-04 |
| Unexpectedly discovered dead body (ever) | 5.07E-04 |
| Head injury ^a^ | 4.73E-04 |
| Encountered land mines ^a^ | 4.69E-04 |
| Sexually assaulted or raped (ever) | 4.06E-04 |
| Enlisted, paygrade E1-E3 | 3.80E-04 |
| Been shot or stabbed | 3.75E-04 |
| Low psychosocial support | 3.72E-04 |
| Age 25-34 | 3.72E-04 |
| Enlisted, paygrade E4-E6 | 3.21E-04 |
| Had a serious operation | 3.14E-04 |
| Some college education | 2.86E-04 |
| $41k-80k income per year | 2.66E-04 |
| Saw dead enemies ^a^ | 2.32E-04 |
| “Other” race (not white, not black) | 1.71E-04 |
| Past-year GAD | 1.30E-04 |
| Child diagnosed with serious illness | 1.13E-04 |
| Hispanic | 8.11E-05 |
| Injured or wounded in combat ^a^ | 7.76E-05 |
| Saw enemies being killed/injured ^a^ | 6.95E-05 |
| Black race | 5.59E-05 |
| Diagnosed with serious illness | 5.42E-05 |
| Childhood sexual abuse | 3.89E-05 |
| Deployed to an area of conflict ^a^ | 7.24E-06 |
| Been kidnapped | -1.75E-06 |
| Killed or think you killed someone in combat ^a^ | -3.06E-05 |
| Close friend sexually assaulted | -5.78E-05 |
| Current or former smoker | -1.17E-04 |
| Been in a fire or explosion | -8.56E-04 |

^a^ During most recent deployment.

PTSD = posttraumatic stress disorder.

GAD = generalized anxiety disorder.

**Table SA3. Mean decrease in accuracy of predictors from 10-fold cross-validated random forest among women with no missing data (n = 251).**

| **Predictor** | **Mean decrease in accuracy** |
| --- | --- |
| Addicted family member | 3.45E-02 |
| Been emotionally mistreated | 2.09E-02 |
| Enlisted, paygrade E1-E3 | 1.86E-02 |
| Current student | 1.78E-02 |
| Age 25+ | 1.36E-02 |
| Close friend injured in [other] accident | 1.19E-02 |
| Low psychosocial support | 1.13E-02 |
| Enlisted, paygrade E4-E9 | 1.13E-02 |
| Childhood (parental) verbal abuse | 1.09E-02 |
| Experienced combat or a war zone | 8.57E-03 |
| Experienced a serious accident/injury (other than car accident) | 7.01E-03 |
| 5+ years of service | 6.66E-03 |
| Childhood physical abuse | 5.15E-03 |
| Deployed to an area of conflict ^a^ | 3.74E-03 |
| Serious transportation accident | 3.47E-03 |
| Been in a fire or explosion | 2.90E-03 |
| Childhood sexual abuse | 2.63E-03 |
| Experienced a natural disaster | 1.46E-03 |
| Been badly beaten up | 1.43E-03 |
| Been exposed to toxic substances | 1.30E-03 |
| Saw dead allies ^a^ | 3.94E-04 |
| Saw wounded civilians ^a^ | 3.31E-04 |
| Saw enemies being killed/injured ^a^ | 2.01E-05 |

^a^ During most recent deployment.

**References for appendix:**

1. King LA, King DW, Vogt DS, Knight J, Samper RE. Deployment Risk and Resilience Inventory: A collection of measures for studying deployment-related experiences of military personnel and veterans. Mil Psychol. 2009;18(2):89–120.

2. Gray MJ, Litz BT, Hsu JL, Lombardo TW. Psychometric properties of the Life Events Checklist. Assessment. 2004 Dec 26;11(4):330–41.

3. Breslau N, Kessler RC, Chilcoat HD, Schultz LR, Davis GC, Andreski P. Trauma and Posttraumatic Stress Disorder in the community. Arch Gen Psychiatry. 1998 Jul 1;55(7):626.

4. Felitti VJ, Anda RF, Nordenberg D, Williamson DF, Spitz AM, Edwards V, et al. Relationship of childhood abuse and household dysfunction to many of the leading Causes of death in adults: The Adverse Childhood Experiences (ACE) Study. Am J Prev Med. 1998 May 1;14(4):245–58.

5. Goldmann E, Calabrese JR, Prescott MR, Tamburrino M, Liberzon I, Slembarski R, et al. Potentially modifiable pre-, peri-, and postdeployment characteristics associated with deployment-related posttraumatic stress disorder among Ohio Army National Guard Soldiers. Ann Epidemiol. 2012 Feb;22(2):71–8.

6. Spitzer RL, Kroenke K, Williams JBW, Löwe B. A brief measure for assessing Generalized Anxiety Disorder. Arch Intern Med. 2006 May 22;166(10):1092.

7. Blanchard EB, Jones-Alexander J, Buckley TC, Forneris CA. Psychometric properties of the PTSD Checklist (PCL). Behav Res Ther. 1996 Aug;34(8):669–73.

8. Sheehan D V, Lecrubier Y, Sheehan KH, Amorim P, Janavs J, Weiller E, et al. The Mini-International Neuropsychiatric Interview (M.I.N.I.): the development and validation of a structured diagnostic psychiatric interview for DSM-IV and ICD-10. J Clin Psychiatry. 1998;59 Suppl 2:22-33;quiz 34-57.

9. Hastie, T., Tibshirani, R., & Friedman J. The elements of statistical learning. 1st ed. New York, NY: Springer; 2001.

10. Morgan J. Technical Report No. 1: Classification and regression tree analysis. 2014.

11. Sutton CD. Classification and regression trees, bagging, and boosting. Handb Stat. 2005;24:303–29.

12. Bi Q, Goodman KE, Kaminsky J, Lessler J. What is machine learning? A primer for the epidemiologist. Am J Epidemiol. 2019 Oct 21;

13. Dasgupta A, Sun Y V., König IR, Bailey-Wilson JE, Malley JD. Brief review of regression-based and machine learning methods in genetic epidemiology: the Genetic Analysis Workshop 17 experience. Genet Epidemiol. 2011;35(S1):S5–11.

14. Strobl C, Malley J, Tutz G. An introduction to recursive partitioning: rationale, application, and characteristics of classification and regression trees, bagging, and random forests. Psychol Methods. 2009 Dec;14(4):323–48.

15. Hastie T, Tibshirani R, Friedman J. Random Forests. In: The Elements of Statistical Learning: Data Mining, Inference, and Prediction. 2nd ed. New York, NY: Springer; 2013. p. 587–603.

16. Liaw A, Wiener M. Classification and regression by randomForest. R News. 2002;2/3:18–22.

17. Shmueli G. To Explain or to Predict? Stat Sci. 2010;25(3):289–310.

18. Geisser S. The predictive sample reuse method with applications. J Am Stat Assoc. 1975;70:320–328.

19. Chen C, Liaw A, Brieman L. Using random forest to learn imbalanced data: Technical Report No. 666. University of California, Berkley. Berkeley, CA; 2004 [cited 2019 Jul 14]. Available from: https://statistics.berkeley.edu/tech-reports/666

20. Breiman L. Random Forests. Mach Learn. 2001;45(5–32):5–30.
